# Assessment of Intima-Media Thickness in Healthy Children Aged 1 to 15 Years

**DOI:** 10.5935/abc.20160030

**Published:** 2016-04

**Authors:** Liz Andréa Villela Baroncini, Lucimary de Castro Sylvestre, Roberto Pecoits Filho

**Affiliations:** Pontifícia Universidade Católica do Paraná, PR - Brazil

**Keywords:** Child, Carotid Artery, Carotid Intima-Media Thickness, Atherosclerosis, Ultrasonography

## Abstract

**Background:**

Carotid intima-media thickness (CIMT) has been shown to be increased in children and adolescents with traditional cardiovascular risk factors such as obesity, hypertension, and chronic kidney disease, compared with those of healthy children.

**Objective:**

To assess the influence of sex, age and body mass index (BMI) on the CIMT in healthy children and adolescents aged 1 to 15 years.

**Methods:**

A total of 280 healthy children and adolescents (males, n=175; mean age, 7.49±3.57 years; mean BMI, 17.94±4.1 kg/m^2^) were screened for CIMT assessment. They were divided into 3 groups according to age: GI, 1 to 5 years [n=93 (33.2%); males, 57; mean BMI, 16±3 kg/m^2^]; GII, 6 to 10 years [n=127 (45.4%); males, 78; mean BMI, 17.9±3.7 kg/m^2^], and GIII, 11 to 15 years [n=60 (21.4%); males, 40; mean BMI, 20.9±4.5 kg/m^2^].

**Results:**

There was no significant difference in CIMT values between male and female children and adolescents (0.43±0.06 mm vs. 0.42±0.05 mm, respectively; p=0.243). CIMT correlated with BMI neither in the total population nor in the 3 age groups according to Pearson correlation coefficient. Subjects aged 11 to 15 years had the highest CIMT values (GI vs. GII, p=0.615; GI vs. GIII, p=0.02; GII vs. GIII, p=0.004).

**Conclusions:**

CIMT is constant in healthy children younger than 10 years, regardless of sex or BMI. CIMT increases after the age of 10 years.

## Introduction

In 1986 Pignoli et al.^1^ and in 2010 O´Leary and Bots,^2^ established B-mode imaging as a useful tool for detecting and monitoring changes in intimal plus medial thickness. This method allows for the evaluation of changes in the arterial wall in areas without localized plaques. Therefore, carotid intima-media thickness (CIMT) measurements have been assessed in several observational and interventional studies. The noninvasive nature of B-mode imaging has made it popular for use in the pre-clinical diagnosis and follow-up of patients with atherosclerosis.^3-5^

The assessment of cardiovascular risk in pediatric patients is challenging. Cardiovascular events or death rarely occur in children, but changes in the cardiovascular system can be identified at an early age in pediatric populations.^6^ CIMT has been shown to be increased in children with traditional cardiovascular risk factors, such as obesity, hypertension, and chronic kidney disease, as compared to healthy children.^7,8^ However, previous studies assessing sex differences in CIMT in healthy pediatric populations have generated conflicting results.^9-11^ These conflicts are probably secondary to the methodologies applied and the fact that the studies included children older than 10 years and adults in the same analyses.^10^

Consequently, the aim of the present study was to evaluate the influence of sex, age, and BMI on CIMT, and to establish parameters for CIMT in healthy children and adolescents aged 1 to 15 years.

## Methods

### Subjects

We selected 280 consecutive healthy Caucasian children and adolescents (males, n=175; mean age, 7.49±3.57 years), who underwent echocardiography for assessment of an innocent cardiac murmur referred by a private pediatrician. The population in the present study was part of the private health care system.

Exclusion criteria were children diagnosed with diabetes, dyslipidemia, hypertension, any systemic disease, and those considered overweight or obese (≥ 85th percentile) for their age.^12,13^

Children were not sedated before the exams. Children who refused to undergo the ultrasound examination and those who did not allow a proper or complete examination, such as very young children, were excluded from the study.

Before the exam, the ultrasonographist collected information on demographic characteristics and cardiovascular risk factors of each parent. Parents were asked about the presence of hypertension, diabetes mellitus, dyslipidemia, coronary artery disease (CAD), and current smoking habit.

Hypertension was defined as a history of treated hypertension. Smoking history was coded as never or current smoker. Subjects were classified as having diabetes when treated for insulin-dependent or non-insulin-dependent diabetes. The use of lipid-lowering drugs was assessed. A history of myocardial infarction, angioplasty or coronary artery bypass graft surgery was recorded, and a positive CAD history was defined as the presence of any of these diseases. Children from parents under treatment for any of these diseases aforementioned were excluded from the study.

The subjects were divided into 3 groups according to age: 1 to 5 years (GI), 6 to 10 years (GII), and 11 to 15 years (GIII). Institutional ethical committee approval was obtained for the study. The legal representative of each child provided written informed consent before examination. Children older than 10 years also signed a consent form.

### Ultrasound measurements

All CIMT measurements were made using high-resolution B-mode ultrasonography (Philips Medical Systems' HD11 platform) with a broadband width linear array transducer L 3-12 MHz. Sonography and readings were conducted by a trained and certified sonographer. The subjects were examined in the supine position with the neck extended and the probe in the anterolateral position. On longitudinal 2D ultrasound images of the carotid artery, the near wall and the far wall are displayed as 2 echogenic lines (the adventitia and intima) that are separated by the hypoechoic media. The distance between the leading edge of the first bright line of the far wall (lumen-intima interface) and the leading edge of the second bright line (media-adventitia interface) is defined as the CIMT.

For this study, we measured the CIMT on the distal 10 mm of the far wall of both the right and left common carotid artery. After zooming and freezing the image, we manually measured the CIMT using electronic calipers. Five measurements were recorded on each side and the average of these measurements was used for the final CIMT analyses.

### Statistical analysis

Quantitative variables are described by mean, median, minimum, and maximum values and standard deviation. Qualitative variables are described as frequencies and percentages. Kolmogorov-Smirnov test was used to test the normality of the distribution. CIMT measurements of both sexes were compared using Student *t* test for independent samples. The age groups were compared using the analysis of variance model with one parameter (ANOVA) and the least significance difference for multiple comparisons. Pearson correlation coefficient was used to evaluate the linear association between CIMT and BMI. Multivariate analysis was performed by adjusting a multiple linear regression model using CIMT as the dependent variable and sex, age, and BMI as independent variables. A p-value < 0.05 indicated statistical significance. The sample size was not calculated at the present study because there are no normative values for CIM in healthy children and adolescents. Data were analyzed with the SPSS v. 20.0 computer program.

## Results

**Figure 1:**
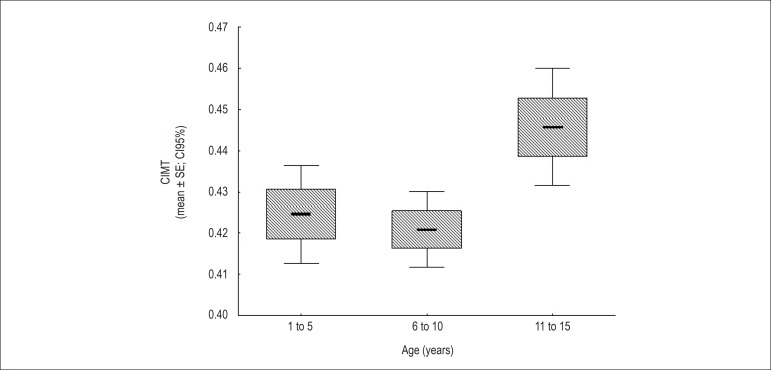
Carotid intima-media thickness (CIMT) among age groups.

This study included 280 healthy children and adolescents (males, n=175; mean age, 7.49±3.57 years; mean BMI, 17.94±4.1 kg/m^2^; mean CIMT, 0.43±0.06 mm). Their characteristics are provided in Table 1. No significant differences in CIMT values were observed between male and female children and adolescents in the total population or among the age groups (Table 2). CIMT was not correlated to BMI in the total population or among the age groups (Table 2). Subjects older than 10 years had the highest CIMT values (Tables 1 and 2, Figure 1).

**Table 1:** General characteristics of the study population

**Groups**	**N (%)**	**Male/Female (n)**	**BMI (kg/m^2^; mean±SD)**	**CIMT (mm; mean±SD)**	*p
GI	93 (33.2%)	57/36	16±3	0.42±0.06	
GII	127 (45.4%)	78/49	17.9±3.7	0.42±0.05	
GIII	60 (21.4%)	40/20	20.9±4.5	0.45±0.05	
Total	280	175/105	17.94±4.1	0.43±0.06	0.013
Groups					†P
GI vs GII					0.615
GI vs GIII					0.02
GII vs GIII					0.004

BMI: body mass index; CIMT: carotid intima-media thickness; SD: standard deviation. GI: 1 to 5 years; GII: 6 to 10 years; GIII: 11 to 15 years.

* Analysis of variance with one parameter, p < 0.05.

† Least significant diference test, p < 0.05.

**Table 2:** Correlations between carotid intima-media thickness (CIMT), sex and body mass index (BMI) among age groups and in the entire study population

**Age (years)**	**Sex**	**N**	**CIMT (mm; mean±SD)**	*P	**†BMI**	**p**
1 a 5	Male	57	0.43±0.06			
	Female	36	0.42±0.05	0.62	0.17	0.11
6 a 10	Male	78	0.42±0.05			
	Female	49	0.41±0.05	0.23	0.01	0.91
11 a 15	Male	40	0.45±0.05			
	Female	20	0.45±0.05	0.98	-0.01	0.92
Total	Male+Female	280			0.11	0.056
	Male	175	0.43±0.06		0.12	0.127
	Female	105	0.42±0.05	0.243	0.10	0.32

SD: standard deviation.

* Student t test for independent samples.

† Pearson correlation coefficient.

## Discussion

Much information is available concerning CIMT in adults, but little information exists regarding CIMT in healthy pediatric populations, despite the need for early detection and prevention of cardiovascular disease.^9^ Most studies of CIMT in pediatric patients have compared healthy children with children who have cardiovascular risk factors, such as hypertension, diabetes, dyslipidemia, obesity, and metabolic syndrome. Additionally, most studies have included subjects aged 10 years or older.^14-16^

In the present study we only included subjects younger than 15 years, and we found that in very young (<10 years old) healthy children, we were unable to detect any significant difference in CIMT when we considered sex and BMI as independent variables. These findings agree with previous studies^2,6-16^ that concluded that the normal carotid arterial wall is unaffected by age or sex until approximately 18 years of age, after which time, there is diffuse progressive intimal thickening. However, we cannot exclude the possibility that our results could be due to the fact that the imaging method used here (high-resolution B mode ultrasonography) is not able to detect such small differences in CIMT due to its low sensitivity. In our study, we confirmed that, as in adults,^17^ CIMT increases with age. These findings could be related to the fact that, by the age of 10, most boys and girls are beginning puberty and undergoing hormonal changes that induce a significant increase in total body fat percentage.^9,18^

Other possible explanation is that CIMT increases as a physiological reaction of the vessel to adapt the age-dependent rise in blood pressure.^6^ In fact, CIMT changes could reflect non-atherosclerotic and adaptive responses to aging and mechanical stress.^6,19^ In the present study, we only included healthy children with normal BMI. CIMT appears to coincide with the normal development of children and increases with age, as it does in adults. Koçyiğit et al.^20^ have studied 91 healthy children aged 7 to 15 years and observed an age-related physiologic thickening of the carotid intima-media that was not related to sex. CIMT is considered a reflection of multiple risk factors, but primary contributors to intima-media thickening are age and hypertension, which do not necessarily reflect the atherosclerotic process.^21-23^ Some studies have corroborated these findings. Lande et al.^14^ have concluded that CIMT is increased in childhood primary hypertension and is independent of the effects of obesity.

Di Pino et al.^24^ have reported that subjects with altered glucose tolerance had associated morphological and functional alterations of the arterial wall; however, these alterations are not likely to be related to hyperglycemia, but, instead, related primarily to aging. Opposing results have also been reported. For example, Stabouli et al.^25^ have studied a similarly aged population and observed that obese children and adolescents have greater CIMT than non-obese subjects, independent of blood pressure. Giannini et al.^26^ have concluded that both obese and thin children present early signs of atherosclerosis, including increased oxidative stress, impaired inflammation, and insulin sensitivity, as well as increased CIMT values.

Pediatric epidemiological studies, as well as case-control and observational studies in children, have confirmed that CIMT is increased in the presence of risk factors such as hypertension, dyslipidemia, diabetes mellitus, and obesity.^15-16,27-29^ Further, traditional cardiovascular risk factors already present in childhood predict the occurrence of preclinical carotid atherosclerosis in adulthood.^30,31^ However, the availability of normative CIMT data for children is limited and most studies have compared CIMT values from children with those from adult populations. Therefore, in the present study, we attempted to assess CIMT in healthy children and adolescents between 1 and 15 years, and to fill a major gap in medical pediatric literature. Our findings could be used to evaluate other children of the same age with comorbidities, such as obesity, hypertension, diabetes mellitus, and dyslipidemia, and children whose parents have cardiovascular risk factors.

## Conclusion

Among healthy children younger than 15 years, there is no significant difference in CIMT between males and females. BMI was not correlated to CIMT in healthy children under the age of 15 years. CIMT is constant in children younger than 10 years, regardless of sex and BMI. CIMT increases after the age of 10 years.
